# The use of nomogram for detecting mild cognitive impairment in patients with type 2 diabetes mellitus

**DOI:** 10.1111/1753-0407.13384

**Published:** 2023-04-13

**Authors:** Rehanguli Maimaitituerxun, Wenhang Chen, Jingsha Xiang, Yu Xie, Atipatsa C. Kaminga, Xin Yin Wu, Letao Chen, Jianzhou Yang, Aizhong Liu, Wenjie Dai

**Affiliations:** ^1^ Department of Epidemiology and Health Statistics, Xiangya School of Public Health Central South University Changsha China; ^2^ Hunan Provincial Key Laboratory of Clinical Epidemiology Changsha China; ^3^ Department of Nephrology, Xiangya Hospital Central South University Changsha China; ^4^ Human Resources Department Central Hospital Affiliated to Shandong First Medical University Jinan China; ^5^ Department of Mathematics and Statistics Mzuzu University Mzuzu Malawi; ^6^ Infection Control Center, Xiangya Hospital Central South University Changsha China; ^7^ Department of Preventive Medicine Changzhi Medical College Changzhi China

**Keywords:** diagnosis, mild cognitive impairment, model, nomogram, type 2 diabetes mellitus, 诊断, 轻度认知障碍, 模型, 列线图, 2型糖尿病

## Abstract

**Background:**

Type 2 diabetes mellitus (T2DM) is highly prevalent worldwide and may lead to a higher rate of cognitive dysfunction. This study aimed to develop and validate a nomogram‐based model to detect mild cognitive impairment (MCI) in T2DM patients.

**Methods:**

Inpatients with T2DM in the endocrinology department of Xiangya Hospital were consecutively enrolled between March and December 2021. Well‐qualified investigators conducted face‐to‐face interviews with participants to retrospectively collect sociodemographic characteristics, lifestyle factors, T2DM‐related information, and history of depression and anxiety. Cognitive function was assessed using the Mini‐Mental State Examination scale. A nomogram was developed to detect MCI based on the results of the multivariable logistic regression analysis. Calibration, discrimination, and clinical utility of the nomogram were subsequently evaluated by calibration plot, receiver operating characteristic curve, and decision curve analysis, respectively.

**Results:**

A total of 496 patients were included in this study. The prevalence of MCI in T2DM patients was 34.1% (95% confidence interval [CI]: 29.9%–38.3%). Age, marital status, household income, diabetes duration, diabetic retinopathy, anxiety, and depression were independently associated with MCI. Nomogram based on these factors had an area under the curve of 0.849 (95% CI: 0.815–0.883), and the threshold probability ranged from 35.0% to 85.0%.

**Conclusions:**

Almost one in three T2DM patients suffered from MCI. The nomogram, based on age, marital status, household income, duration of diabetes, diabetic retinopathy, anxiety, and depression, achieved an optimal diagnosis of MCI. Therefore, it could provide a clinical basis for detecting MCI in T2DM patients.

## Graphical abstract

**Highlights**

- Almost one in three patients with type 2 diabetes mellitus suffered from mild cognitive impairment.
- Age, marital status, household income, diabetes duration, diabetic retinopathy, anxiety, and depression were associated with mild cognitive impairment.
- Nomograms could provide a clinical basis for detecting mild cognitive impairment in patients with type 2 diabetes mellitus.

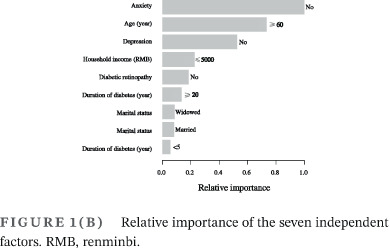

## 摘要

**背景:**2型糖尿病(T2DM)在世界范围内非常普遍, 可导致较高的认知功能障碍发生率。本研究的目的是建立并验证基于列线图的T2DM患者轻度认知功能障碍(MCI)筛查模型。

**方法:**连续纳入2021年3至12月在湘雅医院内分泌科住院的T2DM患者。研究人员对患者进行面对面访谈, 回顾性收集社会人口学特征、生活方式因素、T2DM相关信息以及抑郁和焦虑病史。采用简易精神状态检查量表评估认知功能。根据多因素logistic回归分析结果建立预测MCI的列线图模型。通过校准图、受试者工作特征曲线和决策曲线分析评估列线图的校准度、区分度和临床效用。

**结果:**本研究共纳入496例患者。T2DM患者的MCI患病率为34.1%(95%可信区间:29.9% ~ 38.3%)。年龄、婚姻状况、家庭收入、糖尿病病程、糖尿病视网膜病变、焦虑和抑郁与MCI独立相关。基于这些因素构建的列线图曲线下面积为0.849 (95% CI: 0.815 ~ 0.883), 阈值概率范围为35.0% ~ 85.0%。

**结论:**近1/3的T2DM患者存在MCI。基于年龄、婚姻状况、家庭收入、糖尿病病程、糖尿病视网膜病变、焦虑和抑郁的列线图诊断MCI最佳。列线图可为T2DM患者MCI的检测提供临床依据。

## 1 INTRODUCTION

Diabetes is a serious public health problem globally. According to the latest global diabetes map released by the International Diabetes Federation, approximately 537 million adults are living with diabetes worldwide, and this number is rapidly approaching the prediction level for 2030.^1^ Type 2 diabetes mellitus (T2DM), characterized by elevated blood glucose, insulin resistance, and a relative lack of insulin, accounts for more than 90% of patients with diabetes.^2^ Moreover, accumulating evidence has shown that diabetes is associated with accelerated cognitive decline,^3^, ^4^, ^5^, ^6^ and patients with diabetes have an increased risk of dementia (hazard ratio [HR] = 2.05, 95% confidence interval [CI]: 1.41–2.97) compared with those without diabetes.^7^

Mild cognitive impairment (MCI), generally defined as acquired objective cognitive impairment affecting one or more cognitive domains with largely preserved activities of daily living,^8^, ^9^ is quite prevalent among patients with T2DM.^10^, ^11^, ^12^ It is also recognized as an early stage of dementia.^13^ The presence of MCI in the elderly can lead to a higher conversion rate to dementia.^14^ Previous meta‐analytic studies indicated that the adjusted annual conversion rates from MCI to dementia were 9.6% and 4.9% in hospital‐based and community‐based populations, respectively.^15^ Furthermore, the overall unadjusted pooled odd ratio (OR) for the progression of MCI to dementia in those with diabetes was found to be 1.53 (95% CI: 1.20–1.97).^16^ In addition, the presence of MCI in T2DM patients can lead to reduced diabetes self‐management and poor glycemic control,^8^, ^17^, ^18^, ^19^, ^20^ which may ultimately contribute to poor health‐related outcomes and heavy social and economic burdens.^21^ Therefore, early diagnosis of MCI is crucial for T2DM patients.

Previous studies have shown that socio‐demographic characteristics (e.g. age and educational level), lifestyle factors (e.g. physical activity and smoking status), T2DM‐related characteristics (e.g. duration of T2DM and diabetic complications), and presence of anxiety and depression may be associated with MCI in T2DM patients.^18^, ^22^, ^23^, ^24^, ^25^, ^26^ However, most of these studies have focused exclusively in identifying the biomarkers involved or determining the associated risk factors. Recent studies have shown that nomogram‐based models with individualized, evidence‐based, and highly accurate risk estimation can facilitate management‐related decision making and medical judgments.^27^, ^28^, ^29^ Nevertheless, to date, there is still a lack of optimal models that integrate various risk factors to detect MCI in T2DM patients. Therefore, this study aimed to develop the first nomogram for MCI risk estimation in T2DM patients by comprehensively evaluating the contributions of sociodemographic characteristics, lifestyle factors, T2DM‐related characteristics, depression, and anxiety to the presence of MCI.

## 2 MATERIALS AND METHODS

### 2.1 Ethical approval

The study protocol was approved by the ethics committee of Xiangya School of Public Health, Central South University (No. XYGW–2021–27). Signed informed consents were obtained from all participants.

### 2.2 Study population

Patients hospitalized because of T2DM in the endocrinology department of the Xiangya Hospital, Central South University between March and December 2021 were consecutively included in this study. The inclusion criteria were (a) aged ≥40; (b) met the T2DM diagnostic criteria according to the Chinese Guidelines for the Prevention and Treatment of Type 2 Diabetes (2020 Edition)^30^; and (c) voluntarily participated in this study and signed the informed consent form. Patients with clinical dementia were excluded from this study.

### 2.3 Data collection

Well‐qualified investigators with at least a bachelor's degree in medicine conducted face‐to‐face interviews with participants to retrospectively collect data on sociodemographic characteristics, lifestyle factors, T2DM‐related information, depression, and anxiety. The investigators were blinded to the cognitive function of the participants, which was assessed by experienced physicians.

### 2.4 Outcome variable

The outcome variable for this study was MCI, which was identified using the Mini‐Mental State Examination (MMSE) scale. This scale consisted of 30 items related to attention and orientation, memory, registration, recall, calculation, language, and the ability to draw complex polygons. Its total score ranged from 0 to 30, with higher scores indicating better cognitive function.^31^ Those with a total score of ≤19, ≤ 22, and ≤26 were categorized as MCI for an educational level of illiterate, elementary school, and junior high school or above, respectively, and those with a total score of >19, > 22, and >26 were categorized as normal cognition for an educational level of illiterate, elementary school, and junior high school or above, respectively.^32^

### 2.5 Independent variables

This study considered sociodemographic characteristics, lifestyle factors, T2DM‐related information, depression, and anxiety as independent variables in detecting the outcome variable, which was the presence of MCI.

#### 2.5.1 Sociodemographic and lifestyle factors

Sociodemographic and lifestyle factors included age, sex, ethnicity, marital status, education level, per capita monthly household income (RMB, renminbi), location of residence, current work status, current smoking status, current alcohol consumption, and physical activity intensity.

#### 2.5.2 T2DM‐related information

T2DM‐related information included body mass index (BMI), duration of diabetes, family history of diabetes, diabetes comorbidities (including hypertension, coronary heart disease, chronic kidney disease, stroke, and fatty liver), and diabetes complications (including diabetic foot, diabetic nephropathy, and diabetic retinopathy).

#### 2.5.3 Anxiety and depression

The Hospital Anxiety and Depression Scale (HADS)^33^ was used to measure the severity of anxiety and depression in this study. The scale included two subscales and 14 items, seven of which were for the anxiety subscale (HADS‐A), and the other seven were for the depression subscale (HADS‐D). The total scores of each subscale ranged from 0 to 21, with a total score of ≥8 in the HADS‐A and HADS‐D indicating anxiety and depression, respectively.^34^ The internal consistency Cronbach's alpha coefficients of the HADS‐A and HADS‐D were 0.89 and 0.86, respectively.^35^

### 2.6 Statistical analysis

Continuous variables are described as mean (standard deviation [SD]). They were compared using two independent sample *t*‐tests. Categorical variables are described as frequencies (*n*) and proportions (%). They were compared using the *χ* ^*2*^ test or Fisher's precision probability test. The crude contribution of each independent variable was assessed via univariable logistic regression analyses and quantified by the crude OR and corresponding 95% CI. The independent contribution was further assessed using multivariable logistic regression analysis and quantified using the adjusted OR (aOR) and corresponding 95% CI. A multicollinearity test was performed on these variables, and a variance inflation factor value of less than five indicated no multicollinearity problem.

A nomogram was developed based on the results of multivariable analysis, a graphical representation of the relationship between the observed outcome frequencies and detected probabilities. In a well‐calibrated model, the detection should have fallen on a 45° diagonal line, which was applied with 1000 bootstrap resampling. Detective accuracy (discrimination) was measured using a receiver operating characteristic curve (ROC). The area under the curve (AUC) was calculated to evaluate the classification performance, and a value of 0.7 to 0.9 suggested good accuracy.^36^ The clinical utility of the nomogram was assessed using the decision curve analysis (DCA).^37^ Statistical analyses were performed using the EmpowerStats (www.empowerstats.com) and the R software. All statistical tests were two sided, and a *p* value of <.05 was regarded as statistically significant.

## 3 RESULTS

### 3.1 Participants' characteristics

A total of 530 T2DM patients were initially included in this study, of whom 34 were excluded due to incomplete questionnaire data. Finally, 496 patients were included. The effective response rate was 93.6% (496/530). Among these 496 patients, 169 were categorized as MCI, whereas 327 were categorized as normal cognition based on the MMSE scores. The prevalence of MCI in T2DM patients was 34.1% (95% CI: 29.9%–38.3%).

The characteristics of the study population are summarized in Table 1. Of the 496 patients, 284 (57.3%) were male. The mean age of the patients was 59.6 (9.9) (range: 40–96). Age, marital status, educational level, per capita monthly household income, location of residence, current work, smoking, and alcohol consumption statuses, physical activity intensity, duration of diabetes, and presence of hypertension, coronary heart disease, chronic kidney disease, stroke, fatty liver, diabetic foot, diabetic nephropathy, diabetic retinopathy, anxiety, and depression differed significantly between the MCI group and normal cognition group (*p* < .05).

**TABLE 1:** Characteristics of the study population by mild cognitive impairment (MCI) status.

Factors	Description	Total (*n* = 496, %)	MCI status		*χ* ^*2*^/*t* value	*p* value
			Normal cognition group (*n* = 327, %)	MCI group (*n* = 169, %)		
Age (years)	40–59	271 (54.6)	220 (67.3)	51 (30.2)	61.88	<.001
	≥60	225 (45.4)	107 (32.7)	118 (69.8)		
Sex	Male	284 (57.3)	196 (59.9)	88 (52.1)	2.82	.093
	Female	212 (42.7)	131 (40.1)	81 (47.9)		
Ethnicity	Han	469 (94.6)	307 (93.9)	162 (95.9)	0.84	.358
	Minority	27 (5.4)	20 (6.1)	7 (4.1)		
Marital status	Married	447 (90.1)	2 (0.6)	1 (0.6)	16.39	<.001
	Single	3 (0.6)	302 (92.4)	145 (85.8)		
	Divorced	16 (3.2)	13 (4.0)	3 (1.8)		
	Widowed	30 (6.1)	10 (3.1)	20 (11.8)		
Educational level	Elementary school or below	106 (21.4)	58 (17.7)	48 (28.4)	23.91	<.001
	Middle school	173 (34.9)	101 (30.9)	72 (42.6)		
	High school	101 (20.4)	75 (22.9)	26 (15.4)		
	College or above	116 (23.4)	93 (28.4)	23 (13.6)		
Per capita monthly household income (RMB)	0–5000	341(68.8)	197 (60.2)	144 (85.2)	39.74	<.001
	>5000	155(31.3)	130 (39.8)	25 (14.8)		
Location of residence	Urban area	351 (70.8)	246 (75.2)	105 (62.1)	9.24	.002
	Rural area	145 (29.2)	81 (24.8)	64 (37.9)		
Living alone	Yes	30 (6.1)	15 (4.6)	15 (8.9)	3.61	.060
	No	466 (94.0)	312 (95.4)	154 (91.1)		
Current work status	Employed	145 (29.2)	127 (38.8)	18 (10.7)	42.79	<.001
	Not employed	351 (70.8)	200 (61.2)	151 (89.4)		
Current smoking	Yes	83 (16.7)	64 (19.6)	19 (11.2)	5.55	.019
	No	413 (83.3)	263 (80.4)	150 (88.8)		
Current alcohol consumption	Yes	56 (11.3)	49 (15.0)	7 (4.1)	13.08	<.001
	No	440 (88.7)	278 (85.0)	162 (95.9)		
Physical activity intensity	Low	176 (35.5)	99 (30.3)	77 (45.6)	12.58	.002
	Moderate	270 (54.4)	189 (57.8)	81 (47.9)		
	High	50 (10.1)	39 (11.9)	11 (6.5)		
BMI	<24	269(54.2)	178 (54.4)	91 (53.9)	0.02	.901
	≥24	227(45.8)	149 (45.6)	78 (46.2)		
Duration of diabetes (year)	<5	129 (26.0)	105 (32.1)	24 (14.2)	30.06	<.001
	5–9	95 (19.4)	59 (18.0)	36 (21.3)		
	10–19	196 (39.5)	129 (39.5)	67 (39.7)		
	≥20	76 (15.3)	34 (10.4)	42 (24.9)		
Family history of diabetes	Yes	225 (45.4)	148 (45.3)	77 (45.6)	0.00	.949
	No	271 (54.6)	179 (54.7)	92 (54.4)		
Hypertension	Yes	314 (63.3)	189 (57.8)	125 (74.0)	12.54	<.001
	No	182 (36.7)	138 (42.2)	44 (26.0)		
Hyperlipidemia	Yes	138 (27.8)	91 (27.8)	47 (27.8)	0.00	.997
	No	358 (72.2)	236 (72.2)	122 (72.2)		
Coronary heart disease	Yes	92 (18.6)	45 (13.8)	47 (27.8)	14.56	<.001
	No	404 (81.5)	282 (86.2)	122 (72.2)		
Chronic kidney disease	Yes	165 (33.3)	97 (29.7)	68 (40.2)	5.61	.018
	No	331 (66.8)	230 (70.3)	101 (59.8)		
Stroke	Yes	71 (14.3)	36 (11.0)	35 (20.7)	8.55	.004
	No	425 (85.7)	291 (89.0)	134 (79.3)		
Fatty liver	Yes	117 (23.6)	90 (27.5)	27 (16.0)	8.24	.004
	No	379 (76.4)	237 (72.5)	142 (84.0)		
Diabetic nephropathy	Yes	262 (52.8)	150 (45.9)	112 (66.3)	18.61	<.001
	No	234 (47.2)	177 (54.1)	57 (33.7)		
Diabetic retinopathy	Yes	226 (45.6)	133 (40.7)	93 (55.0)	9.26	.002
	No	270 (54.4)	194 (59.3)	76 (45.0)		
Diabetic foot	Yes	49 (9.9)	24 (7.3)	25 (14.8)	6.95	.008
	No	447 (90.1)	303 (92.7)	144 (85.2)		
Anxiety	Yes	108 (21.8)	32 (9.8)	76 (45.0)	80.98	<.001
	No	388 (78.2)	295 (90.2)	93 (55.0)		
Depression	Yes	135 (27.2)	49 (15.0)	86 (50.9)	72.50	<.001
	No	361 (72.8)	278 (85.0)	83 (49.1)		

Abbreviations: BMI, body mass index; RMB, renminbi.

### 3.2 Univariable and multivariable analyses

The results of the univariable and multivariable logistic regression analyses are presented in Table 2. Those aged ≥60 (aOR = 4.08, 95% CI: 2.21–7.54), widowed participants (aOR = 2.92, 95% CI: 1.13–7.52), those with a duration of diabetes of 5–9 years (aOR = 2.20, 95% CI: 1.01–4.78) or ≥ 20 years (aOR = 2.29, 95% CI: 1.01–5.23), those with diabetic retinopathy (aOR = 1.78, 95% CI: 1.05–3.02), and those with anxiety (aOR = 3.05, 95% CI: 1.60–5.82) or depression (aOR = 2.37, 95% CI: 1.30–4.32) were at higher risk of MCI. Furthermore, those with a per capita monthly household income of >5000 RMB (aOR = 0.47, 95% CI: 0.24–0.93) were at lower risk of MCI.

**TABLE 2:** Univariable and multivariable logistic regression analyses on factors to detect mild cognitive impairment (MCI).

Factors	Description	Univariable logistic regression		Multivariable logistic regression	
		Crude OR (95% CI)	*p* value	Adjusted OR (95% CI)	*p* value
Age (years)	≥60	4.76 (3.18–7.11)	<.001	4.08 (2.21–7.54)	<.001
Marital status	Single	1.04 (0.09–11.58)	.974	1.81 (0.01–226.04)	.810
	Divorced	0.48 (0.13–1.71)	.259	0.26 (0.05–1.43)	.122
	Widowed	4.17 (1.90–9.13)	.000	2.92 (1.13–7.52)	.027
Educational level	Junior high school	0.86 (0.53–1.40)	.549	1.66 (0.86–3.18)	.129
	High or secondary school	0.42 (0.23–0.75)	.004	0.79 (0.36–1.74)	.562
	College or above	0.30 (0.16–0.54)	<.001	1.12 (0.46–2.70)	.807
Per capita monthly household income (RMB)	>5000	0.26 (0.16–0.42)	<.001	0.47 (0.24–0.93)	.031
Location of residence	Rural area	1.85 (1.24–2.76)	.003	1.53 (0.85–2.73)	.154
Current work status	Not employed	5.33 (3.11–9.11)	<.001	1.30 (0.64–2.65)	.466
Current smoking	Yes	0.52 (0.30–0.90)	.020	0.95 (0.43–2.10)	.907
Current alcohol consumption	Yes	0.25 (0.11–0.55)	.001	0.45 (0.15–1.30)	.140
Physical activity intensity	Moderate	0.55 (0.37–0.82)	.003	0.81 (0.47–1.40)	.444
	High	0.36 (0.17–0.75)	.007	0.48 (0.19–1.20)	.116
Duration of diabetes (years)	5–9	2.67 (1.45–4.90)	.002	2.20 (1.01–4.78)	.047
	10–19	2.27 (1.33–3.87)	.003	1.12 (0.57–2.19)	.749
	≥20	5.40 (2.87–10.18)	<.001	2.29 (1.01–5.23)	.049
Hypertension	Yes	2.07 (1.38–3.12)	.001	1.09 (0.62–1.93)	.756
Coronary heart disease	Yes	2.41 (1.52–3.83)	.000	1.30 (0.70–2.41)	.410
Chronic kidney disease	Yes	1.60 (1.08–2.35)	.018	0.95 (0.50–1.81)	.876
Stroke	Yes	2.11 (1.27–3.51)	.004	1.35 (0.70–2.63)	.372
Fatty liver	Yes	0.50 (0.31–0.81)	.005	0.62 (0.33–1.17)	.139
Diabetic nephropathy	Yes	2.32 (1.58–3.41)	<.001	1.53 (0.82–2.89)	.185
Diabetic retinopathy	Yes	1.78 (1.23–2.60)	.003	1.78 (1.05–3.02)	.033
Diabetic foot	Yes	2.19 (1.21–3.97)	.010	1.77 (0.81–3.89)	.153
Anxiety	Yes	7.53 (4.69–12.11)	<.001	3.05 (1.60–5.82)	.001
Depression	Yes	5.88 (3.83–9.02)	<.001	2.37 (1.30–4.32)	.005

Abbreviations: CI, confidence interval; OR, odds ratio; RMB, renminbi.

### 3.3 Development of the MCI detective nomogram

**FIGURE 1:**
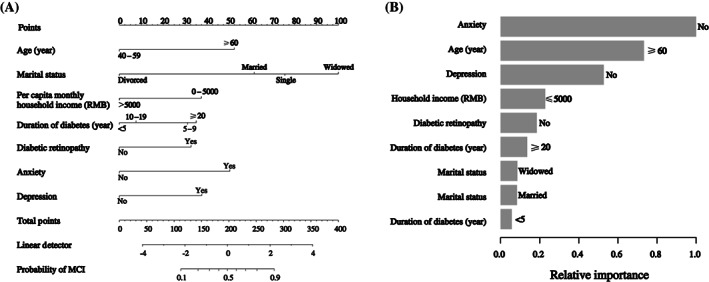
(A) Visualization nomogram to detect mild cognitive impairment (MCI) in type 2 diabetes mellitus (T2DM) patients. First, a standard line marked with 0–100 points is drawn from the points axis. Each of the seven detective factors (age, marital status, household income, duration of diabetes, diabetic retinopathy, anxiety, and depression) corresponds to a parameter value by drawing a visualization line graph from the point axis. Finally, a line from the total‐points axis, which is the sum of the points for the seven detective factors in the range of 0 to 400, is below the last variable point axis. (B) Relative importance of the seven independent factors. RMB, renminbi.

A nomogram was developed based on age, marital status, per capita monthly household income, diabetes duration, diabetic retinopathy, anxiety, and depression. A standard scoring system was established according to the aOR values of these seven factors, and the score of each detection factor for MCI was evaluated. The probability of detecting MCI was effectively estimated by adding the scores of these seven factors (Figure 1A). The relative importance of each factor is shown in Figure 1B.

### 3.4 Validation of the nomogram

**FIGURE 2:**
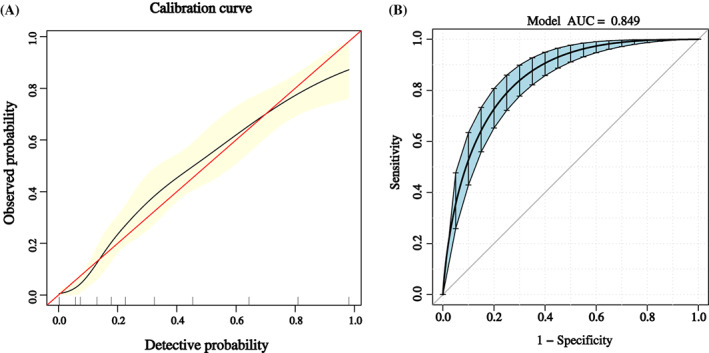
(A) Calibration plot of the nomogram. The detective performance for mild cognitive impairment (MCI) was evaluated by 1000 bootstrap resampling. The detective probability and the observed probability are presented in the *x* and *y* axes, respectively. (B) The receiver operating characteristic curve of the nomogram. The sensitivity and specificity of this curve reflect the discrimination of the nomogram for detecting MCI. AUC, area under the curve; RMB, renminbi.

The calibration curve of the nomogram is shown in Figure 2A, which suggested good agreement between the constructed model and true observations. The ROC curve is shown in Figure 2B. The AUC value was 0.849 (95% CI: 0.815–0.883), which indicated good accuracy.

### 3.5 Clinical utility of the nomogram

**FIGURE 3:**
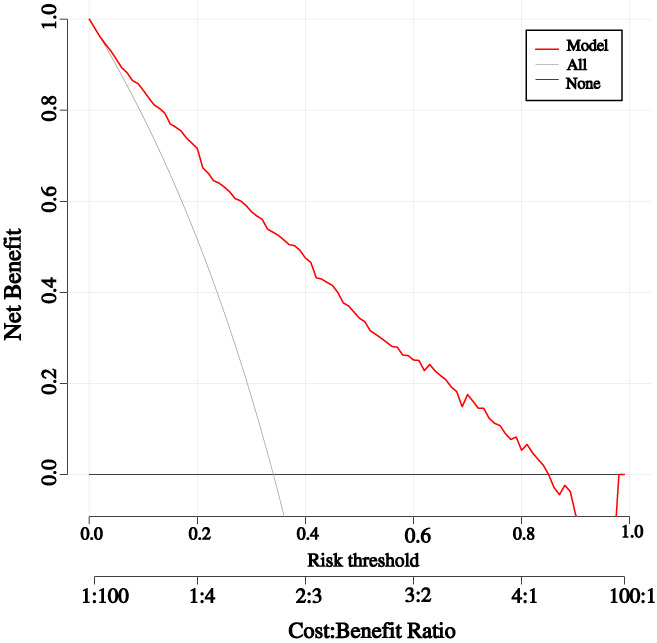
The decision curve analysis of the nomogram. The *x* and *y* axes represent the threshold probability and net benefit, respectively.

The threshold probability of the nomogram ranged from 35.0% to 85.0% by DCA. The clinical utility of the nomogram is shown in Figure 3.

## 4 DISCUSSION

Previous studies have identified several risk factors or biomarkers for MCI in T2DM patients.^22^, ^23^ This study developed and validated a nomogram‐based model to detect MCI in T2DM patients by considering seven factors including age, marital status, household income, duration of diabetes, prediabetic retinopathy, anxiety, and depression. The present nomogram had good discrimination and calibration, as well as satisfactory clinical utility, which could facilitate the detection of MCI in T2DM patients by decision makers.

This study utilized the MMSE to assess the participants' cognitive function and found that the prevalence of MCI in T2DM patients was 34.1% (95% CI: 29.9%–38.3%). The Montreal Cognitive Assessment (MoCA) and MMSE are two of the most commonly used tools for assessing MCI worldwide,^38^, ^39^ and a high degree of consistency between these two scales has been observed in T2DM patients.^40^, ^41^ For example, in a prior meta‐analytic study estimating the pooled prevalence of MCI in T2DM patients, it was found that 9 of the 12 eligible studies used the MMSE or MoCA to assess the presence of MCI.^40^ Specifically, the estimated pooled prevalence derived from MMSE and MoCA was 49.9% (95% CI: 35.1%–64.8%) and 46.6% (95% CI: 34.3%–58.8%), respectively.^40^ Nevertheless, some studies suggested that MCI prevalence was higher using MoCA than MMSE in middle‐aged and older Chinese populations.^42^ Therefore, more studies which diagnose MCI using the Peterson criteria are warranted in order to obtain a more reliable estimate of MCI prevalence among T2DM patients.^43^

In relation to sociodemographic characteristics, older age has been a well‐known risk factor for both MCI and T2DM,^17^, ^44^ and the increased risk of MCI with older age has also been observed in this study, with those aged ≥60 at a 4.08‐fold risk of MCI. This can be explained by the fact that brain function declines with increasing age.^45^ In addition, this study found that widowed participants were at a higher risk of MCI, which was consistent with the findings of previous studies.^46^, ^47^, ^48^ Specifically, Chen et al^47^ found that divorced, separated, and widowed individuals showed more cognitive impairment, and Xu et al^48^ found that being single was associated with lower MMSE scores. Furthermore, household income has been found to be associated with both cognitive function and diabetes. In fact, it has been utilized as an indicator in various socioeconomic studies.^49^, ^50^, ^51^ Consistently, this study found that those with a per capita monthly household income of >5000 RMB had a lower risk of MCI than their counterparts. Based on these findings, it was highly recommended that health care providers allocate more cognitive intervention resources to T2DM patients aged ≥60, those with a lower household income, or widowed individuals.

The duration of diabetes can predict the effectiveness of intensive glucose control and self‐management education programs.^52^, ^53^ Its association with cognitive function remains controversial in T2DM patients. Specifically, Roberts et al^54^ reported that a longer duration of T2DM was associated with higher risk of MCI, whereas Reinke et al^55^ reported a U‐shaped association between T2DM duration and risk of dementia. This study found that, compared to those with T2DM duration of <5 years, those with a T2DM duration of 5–9 years and ≥ 20 years had a 2.20‐ and 2.29‐fold risk of MCI, respectively. However, the risk of MCI did not differ significantly between those with a T2DM duration of <5 years and those with a T2DM duration of 10–19 years, which might be explained partly by the low statistical power when this association was estimated. Therefore, further longitudinal research with a large sample size is still needed to identify the relationship between duration of diabetes and MCI in T2DM patients.

Diabetic retinopathy is one of the most common complications of T2DM and is correlated with cognitive function in T2DM patients.^56^ Crosby‐Nwaobi et al^57^ found that the association between diabetic retinopathy and retinal microvasculature disease was potentially an evidence of a microvascular component of cognitive impairment. Additionally, a population‐based cohort study by Gupta et al^58^ reported that diabetic retinopathy, particularly at the more severe stage, was associated with an increased risk of developing cognitive impairment. Similarly, this study found that diabetic retinopathy was associated with a higher risk of MCI in T2DM patients. Therefore, special attention should be paid to those with diabetic retinopathy in the clinical practice.

Anxiety and depression are two of the most common mental health concerns among T2DM patients,^59^, ^60^, ^61^ and prior studies have linked these conditions to worse cognitive function.^25^, ^62^ For example, a recent systematic review and meta‐analysis involving 10 eligible studies showed that depression was associated with worse cognitive function and greater dementia risk.^63^ Additionally, the role of anxiety in the risk of dementia has been identified in some meta‐analyses.^64^, ^65^ Consistently, this study found that anxiety and depression were both independently associated with a higher risk of MCI in T2DM patients, highlighting the importance of regular screening for anxiety and depression in T2DM patients to maintain cognitive function.

In summary, this study developed and validated a visualized nomogram to detect MCI in T2DM patients. The use of a nomogram in evaluating the risk of MCI in T2DM patients is a new concept that can potentially yield reliable optimal models as guides for better clinical decision‐making and administration of individualized interventions. However, this study has some limitations. First, the nomogram developed in the current study was internally validated using bootstrap validation without external datasets. Second, several information in this study, such as duration of diabetes, was collected retrospectively; hence, recall bias may have existed. Lastly, whether these findings can be generalized to other populations remains unclear.

## FUNDING INFORMATION

This study was supported by the National Natural Science Foundation of China (82103939), the National Natural Science Foundation of Hunan Province (2021JJ40805), the start‐up research fund of Central South University (202044003), and the National Key R&D Program of China (2020YFC2008600).

## CONFLICT OF INTEREST STATEMENT

The authors report there are no competing interests to declare.
